# A Treatise on General Pathology

**Published:** 1853-08

**Authors:** 


*A Treatise on General Pathology.* By Dr. J. Henle, Prof. of Anatomy and Physiology in Heidelburg. Translated from the German, by Henry C. Preston, A. M., M. D. Philadelphia: Lindsay & Blakiston. 1853. 8vo, pp. 391.

We had noticed in several of our exchanges, announcements of the publication of this work, and anticipated much gratification from its perusal. Two or three attempts, however, have led us to postpone the undertaking, if not indefinitely, at least till after the summer solstice. The translator asks the indulgence of the reader for any imperfections in the English version, and suggests that "those who have never attempted the difficult task of translating a metaphysical German work into good English, are not the most competent judges of the manner of its execution." We confess that we are not sufficiently conversant with the German language and literature to know how much of our disappointment to charge upon the translator, and how much on the author; but this we do know, the work, as it is presented to us, is in not very intelligible English. In the selection of words, and the construction of sentences, it almost seems at times as if a studied attempt had been made to annoy and bewilder the reader. We are quite willing to admit that a share of the difficulty rests with ourselves; and we have no wish that others should be discouraged by our ill-success. We must leave the task, for the present, of reviewing the book to those whose facility of comprehension, and patience, enable them, more readily than ourselves, to master its contents.

It is simple justice to the worthy publishers to say, that the getting up of the work is excellent.

